# From the Semen Collection Method to the Hatchlings: The Use of Cryopreserved Sperm from Pheasants Fed an Antioxidant-Enriched Diet

**DOI:** 10.3390/ani11092624

**Published:** 2021-09-07

**Authors:** Annelisse Castillo, Carla Lenzi, Andrea Pirone, Alessandro Baglini, Claudia Russo, Dominga Soglia, Achille Schiavone, Margherita Marzoni Fecia di Cossato

**Affiliations:** 1 Dipartimento di Scienze Veterinarie, Università Degli Studi di Torino, Largo Paolo Braccini 2, 10095 Grugliasco, TO, Italy; annelisse.castillogarrido@unito.it (A.C.); dominga.soglia@unito.it (D.S.); 2 Dipartimento di Scienze Veterinarie, Università di Pisa, Viale Delle Piagge 2, 56124 Pisa, PI, Italy; carla.lenzi@unipi.it (C.L.); andrea.pirone@unipi.it (A.P.); alessandro.baglini@unipi.it (A.B.); claudia.russo@unipi.it (C.R.); margherita.marzoni@unipi.it (M.M.F.d.C.)

**Keywords:** pheasant semen, freezing process, vitamin E, selenium, AI, hatchability

## Abstract

**Simple Summary:**

Although cryopreservation techniques for use in bird species have advanced greatly over recent decades, especially in relation to domestic species, major gaps in our knowledge and technical capacities remain due to the complexity of the process and the unique particularities of sperm from different species. The hatchability of chicks is the decisive parameter that demonstrates the quality of a frozen–thawed sperm. Since very little information has been published about the common pheasant, a total of six artificial inseminations (AIs) were performed at 3–4-day intervals with doses of 35 × 10^6^ of normal live thawed sperm on a total of 40 females. The inseminated sperm were collected from pheasants fed either a basal diet or an antioxidant-enriched diet and were then processed using a pellet freezing–thawing method, in which dimethylacetamide was used as a cryoprotectant. Regardless of the male birds’ dietary group, the resulting fertility rate from frozen–thawed sperm was approx. 30%, with 8–9 chicks hatching for every 100 eggs incubated.

**Abstract:**

A widely used approach to preserving genetic diversity in birds involves the cryopreservation of semen. In this process, cells are subjected to physical and chemical stresses, but not all cell species respond equally. Many studies have been published on the freezing–thawing of sperm cells from a wide variety of domestic and wild species, on issues ranging from the sperm quality to different protocols, fertilisation success rates, etc. Nevertheless, very little information is available on the common pheasant. To fill this gap, the aim of this study was to describe the pheasant semen collection method, evaluate some qualitative parameters of sperm from males fed an antioxidant-enriched diet, and to test the in vivo fertilising capacity of the cryo-preserved semen. The freezing protocol employed involved pellets thawed by the hotplate method. Dimethylacetamide was used as a cryoprotectant at a final concentration of 6%. A total of six AIs were performed at 3-4-day intervals on a total of 40 females with doses of 35 × 10^6^ of normal live thawed sperm. Males receiving the enriched diet produce more abundant and concentrated ejaculates. Freeze–thawed sperm lost 85% of their initial mobility, and diet influenced neither sperm mobility nor viability. The enriched diet did improve the number of normal freeze–thawed cells and was associated with a lower sperm fracture incidence. Regardless of the dietary group, frozen–thawed sperm resulted in a fertility rate of 30%, with 8-9 chicks hatching for every 100 eggs incubated.

## 1. Introduction

Since the first successful attempt to cryopreserve chicken sperm more than seventy years ago, many studies have been performed to improve this reproductive technology [1]. Sperm cryopreservation has been investigated in many domestic bird species, including, for example, turkeys, ducks [2], geese [3], guinea fowl [4], quail [5] and pigeons [6]. The literature now documents sperm cryopreservation protocols for a number of bird species used in animal farming as well as fancy species [7,8]. The success of the cryopreservation of bird semen depends on several factors, including bird species, breed, collection method, dilution and diluent, sperm concentration, cooling rate, cryoprotectant and the freezing and thawing methods employed [7,8].

Bird sperm are highly differentiated cells, the job of which it is to fertilise the oocyte and pass on their genetic information. They possess a low cytoplasm content and a large cellular membrane [9]. The cell membrane plays an important role in sperm maturation and in the egg fertilisation process, in which the cell undergoes a series of biochemical and functional changes [10]. Indeed, the process of fertilisation requires a membrane with specific adaptations at its points of fusion with the oolemma and in its sperm organelles such as, for example, the acrosome, to prepare it for fertilisation [9]. The sperm membrane is characterised by its permeability, fluidity and lipid content [9,10,11], which is high in phospholipids and sterols [12], mainly in polyunsaturated fatty acids [13]. This feature renders the cell membrane of avian sperm highly vulnerable to lysis and lipid peroxidation. Avian semen naturally contains antioxidants and enzymatic defences that protect the cell structure [14]. Stress factors acting on semen during manipulation, liquid storage or cryopreservation endanger and compromise sperm integrity. Therefore, it is necessary to offer the bird additional molecules and/or mineral trace elements to sustain a highly efficient antioxidant system, which consequently diminishes the damage incurred during the freezing/thawing process and artificial insemination (AI) [10]. Strategies for protecting sperm against excessive reactive oxygen species (ROS) production during cryopreservation can be carried out by dietary means [13,15,16] or by supplementing the freezing medium with antioxidants [17,18,19], similarly to protocols used for mammals, for example, rabbit [20] or bull [21].

Cell antioxidant systems include natural fat-soluble antioxidants such as vitamin E, and antioxidant enzymes are also involved, such as superoxide dismutase and glutathione peroxidase; selenium is an integral cofactor of the latter enzyme [13]. Moreover, the interaction between vitamin E and selenium may increase glutathione peroxidase production [13,22]. It is well established that dietary supplementation with selenium, vitamin E and carotenoids can modulate the antioxidant defences in poultry [13].

Among game birds, the common pheasant represents one of the most popular and economically important species, kept mainly for hunting purposes. Moreover, its eggs and meat offer a valuable food source to the human diet [23,24]. Breeding programs are followed either to increase the productive performance of populations bred for their meat or for hunting purposes, and to maintain specific traits, mainly those found in wild populations for genetic conservation and release into the wild [23]. Sperm cryopreservation provides a key tool for supporting the execution of these programs by helping to spread genetic diversity and through its contribution to the development of reproductive technologies [9,25]. To date, very few studies have been reported on the cryopreservation of semen from the common pheasant [26,27,28].

Semen collection in birds is traditionally performed by the dorso-abdominal massage technique, as first described more than 80 years ago [29]. The collection method largely influences the quality of the collected semen. The ability to avoid contaminants in the semen and to minimise handling stress to the birds are two fundamental aspects of a collection technique [30,31,32,33,34]. Therefore, semen collection should be considered the first essential factor for the cryopreservation process and successful AIs. This aspect was recently highlighted in relation to chickens, for which the number of penetrating sperm, evaluated using the inner perivitelline membrane penetration assay, was unexpectedly greater in frozen–thawed sperm compared with fresh sperm, indicating a better performance in the cryopreserved sperm [35]. These authors attributed this result to the benefits obtained from the training of male: while the fresh sperm were obtained from males who were also allowed to mate naturally, and only occasionally subjected to massage collection, the cryopreserved sperm came from roosters kept exclusively as donors, and were thus habituated to the massage collection [35]. The aim of the present study was to describe the pheasant semen collection technique, to evaluate some qualitative parameters of sperm from males fed an antioxidant-enriched diet, and to test the fertilising capacity of the cryo-preserved semen in vivo.

## 2. Materials and Methods

### 2.1. Reagents

All chemicals were purchased from Sigma Aldrich (Milano, Italy), with the exception of Accudenz, a cell separation media that was purchased from Accurate Chemical and Scientific Corp. (Westbury, NY, USA).

### 2.2. Birds

Thirty male common pheasants (*Phasianus colchicus mongolicus*) were housed for their first reproductive season in a peaceful location far from public roads in open-air aviaries containing perches; each male was kept alone in a 6 m^2^ allocated space [36]. Birds were randomly divided into 2 groups and fed ad libitum one of two diets which differed according to their α-tocopheril-acetate (Vit. E) and selenium contents: the control group (CON) diet constituted a basal commercial feed (11.51 MJ/kg of M.E., 19% of C.P., 1% fish oil; plus 40 mg α-tocopheril-acetate and 0.1 mg selenomethionine per kg of feed); the antioxidant-enriched group (E-Se) diet was fed the same basal commercial feed supplemented with additional α-tocopheril-acetate and selenium to a final concentration of 200 mg α-tocopheril-acetate and 0.3 mg selenomethionine per kg of feed. All pheasants received the diets for one month. Semen was collected twelve times (every 3–4 days) over a 39-day period (May–June).

### 2.3. Semen Collection and Semen Quality Parameters

Semen was collected by means of the dorso-abdominal massage technique by two operators in the following way: one operator captures the bird with a net to minimise potential stress [36]. The operator then assumes a sitting position, placing the bird with its abdomen on his lap, such that the bird’s legs are dangling downward. At this point, the second operator gently restrains the bird by placing the animal’s legs through the circle of space created by touching his thumb and middle finger together. In this way, the bird is able to move forward and backwards its legs. The first operator can then use both hands to stimulate the bird. The massage initiates from the bird’s back, stroking the bird with one hand starting from behind the level of wing attachment and moving backwards towards the base of the tail. This movement is performed 3 or 4 times. The bird responds immediately by lifting his tail. This movement is repeated a second time, but with the second hand under the cloaca. This is repeated 2 to 3 times, at this point the bird everts the cloaca and ejaculates voluntarily. The only intervention made by the handler is to prolong the bird’s eversion for a few seconds to facilitate the second operator who collects the semen. The second operator collects the semen by means of an aspirating device. During this process, the bird can move its wings and legs as he desires. The objective is to permit the bird as much liberty of movement as possible, such that he feels comfortable and not constrained.

Semen was collected from each male and directly introduced into the collection tube with 50 µL of pre-freezing Lake’s diluent [37] plus 50 mM glycine diluent (except in tubes destined for pH measurements). Immediately thereafter, it was placed inside a portable refrigerator set to 18 °C until all donations had been collected. Within 40 min from collection, clean ejaculates were chosen, and semen was pooled according to the diet into two samples (CON and E-Se). Ejaculate volume was assessed by weighing the tubes, before and after collection (Sartorius BL 150S, ±0.001 g). The pH was measured for three random samples without diluent for three non-consecutive days of collection (Hamilton 238140, Hanna Instruments, Italy). Sperm concentration was assessed in duplicate using a Bürker–Türk counting chamber (in a 5% formalin and 0.9% NaCl solution). The sperm viability percentage and sperm morphology were evaluated in triplicate in samples of 500 cells using the eosin-nigrosin staining technique [38]. Viable cells did not stain at all, whereas cells considered dead appeared totally or partially stained pink. The sperm viability percentage was calculated relative to total counted sperm (V-CS). From the live sperm sample, abnormal cells were grouped according to anomalies of the head or the tail. The percentage of normal sperm was calculated relative to total live sperm (N-LS). The percentages of abnormal heads (Ah-LS) and abnormal tails (At-LS) were calculated relative to total live sperm. Spermatozoa mobility (SM) was assessed in triplicate using Accudenz methodology [39], which measures the ability of these cells to penetrate a viscous medium at a temperature of 41 °C, and this penetration was measured by spectrophotometry at an absorbance of 550 nm.

### 2.4. Freezing Methodology in Pellets

Simultaneously to the assessment of the semen quality parameters of a pool sample (900 µL), pools were divided into 300 µL aliquots and processed for the freezing procedure into pellets [40] adapted for pheasants [28,41]. Briefly, samples were diluted 1:3 (*v*:*v*) in pre-freezing Lake’s diluent [37] supplemented with 50 mM glycine, and rapidly cooled inside a device set to −6 °C until the sample temperature indicated 5 °C. Samples were left for 10 min at 5 °C, dimethylacetamide cryoprotectant (DMA) added to obtain a final concentration of 6%, mixed manually for one minute, left to stabilise for 4 min, and then 100 µL semen aliquots were dropped directly into liquid nitrogen. The resulting frozen semen pellets were collected and stored inside cryovials in liquid nitrogen for one year.

AIs were performed during the successive pheasant reproductive season. Pellets were melted employing the hotplate method at 75 °C; one at a time, the pellets were placed on an aluminium dish and pushed gently using a micropipette tip in order to collect the melted semen quickly [41].

### 2.5. Thawed Semen Quality

Immediately after the semen was thawed, the qualitative parameters were evaluated following the same protocols previously described for fresh semen. The percentage of viable thawed cells was calculated considering the total number of counted thawed sperm as 100% (V-CTS). The mobility results are reported as percentages with respect to fresh sperm cell mobility (SM-FS). The percentage of normal cells was calculated considering the total number of thawed live sperm as 100% (N-LTS). The viability rate, mobility and percentage of normal cells were compared between fresh and frozen–thawed (F–T) sperm. Within the portion of live F–T sperm, cells were grouped according to the presence of lesions of the head or the tail. The percentages of abnormal thawed heads (Ah-LTS) and abnormal thawed tails (At-LTS) were calculated relative to total live thawed sperm. The head injuries identified were: bent, fractured, coiled, swollen-detached, knotted, or headless. The tail injuries identified were: looping, fractured, and coiled. Data are reported as percentages relative to total live thawed sperm (LTS).

### 2.6. Females and Artificial Inseminations

Forty common female pheasants (*Phasianus colchicus mongolicus*) in their first reproductive season were housed in a peaceful location far from public roads in open-air sandy aviaries containing perches; each female was provided with a 3.6 m^2^ space [36]. Two weeks prior to commencing the AIs, all females were fed ad libitum on a breeders commercial feed (11.51 MJ/kg of M.E., 19% of C.P.). The massage method applied to females was substantially the same as described above for male birds, with changes related to the insemination. Additionally, in this case, the eversion of the cloaca was exclusively carried out by the bird; no force was applied by the operator. Vaginal insemination was performed by means of an adapted Gilson pipette (Gilson, Pipetman P200, H23601S64092K) for bird insemination (IMV Technologies, L’Aigle, France). During the release of semen, the female performs cloacal movements, thus taking up the inseminated dose.

The fertilising ability of the F–T semen collected the previous year was evaluated through AIs performed on forty females, divided randomly into two groups according to the bird group from which the inseminated pellets were generated: the control group (CON) and α-tocopheril-acetate-selenium group (E-Se). All females received 35 × 10^6^ normal live thawed sperm in each insemination. Inseminations were performed on six days, designated days 1, 2, 6, 9, 13, and 16. Egg fertility was determined on eggs collected from day 2 to day 21 after the first AI. Eggs were weighed daily and set the day after laid. They were candled on day 7 of incubation, and those judged as infertile were broken up for macroscopic examination of the germinal disc. Egg laying, fertility and hatchability were calculated according to the following formulas:

Egg laying% = (total laid eggs/females * days) * 100(1)

egg fertility% = (total fertile eggs/total incubated eggs) * 100(2)

hatchability% = (total hatched chicks/total fertile eggs) * 100(3)

### 2.7. Statistical Analysis

One way ANOVA was applied by the GLM procedure using SAS software (SAS Studio, v. 3.8) to evaluate the qualitative parameters of fresh and thawed semen and egg parameters. The correlation between the semen qualitative parameters was evaluated by the CORR procedure (SAS Studio, v. 3.8). The statistical model considered the diet as the fixed effect. Percentage data were normalised through √x Arcsine transformation. A *p* < 0.05 was considered statistically significant.

## 3. Results

The sperm qualitative indicators in the fresh and F–T semen from the two pheasant groups fed the control diet (CON group) vs. the diet enriched with α-tocopheril-acetate and selenomethionine (E-Se group) are reported in Table 1. Ejaculate volume and sperm concentration in the fresh semen were positively affected by diet enrichment (*p* < 0.01). The males in the E-Se group produced more abundant ejaculates, which were a third larger in volume compared with those from the CON group. Moreover, the E-Se group ejaculates had a higher sperm concentration (1.63 × 10^9^ more cells/mL).

In the sperm subjected to the F–T procedure, a higher number of normal cells was observed in the E-Se group (+17%), and the rate of abnormal heads and tails was higher in the CON group than in the E-Se group (*p* < 0.01). The viability rate, mobility, and percentage of normal cells were significantly different between fresh and F–T sperm, as expected (*p* < 0.01). The number of sperm in F–T samples from the CON group was 40% lower with respect to the number of fresh sperm from these birds, whereas the same comparison in the E-Se group gave a negative difference of 29%. The viability of F–T sperm was 25% in both dietary groups; the reduction in F–T sperm mobility was 84% in the CON group and 82% in the E-Se group with respect to the fresh material. Fractures to tails and heads were the most frequent injuries detected in both groups, and both were significantly higher in the CON group (*p* < 0.01). Looping tails and bent heads were the next most common injuries detected in both dietary groups.

Table 2 reports the correlations between the qualitative parameters in the fresh sperm. The CON group fresh sperm shows positive correlations between pH and sperm concentration (SC; *p* < 0.01), N-LS (*p* < 0.01), At-LS (*p* < 0.01) and Ah-LS (*p* < 0.01). Positive correlations also exist between SC and N-LS (*p* < 0.05) and between SC and Ah-LS (p < 0.05), as well as between At-LS and N-LS (*p* < 0.01) and between Ah-LS and N-LS (*p* < 0.01) and At-LS (*p* < 0.01). In the E-Se sperm group, positive correlations are observed between Ah-LS and pH (*p* < 0.01), N-LS (*p* < 0.01) and At-LS (*p* < 0.01). Positive correlations are also observed between At-LS, pH (*p* < 0.05) and N-LS (*p* < 0.05).

Table 3 reports the correlations between the qualitative parameters in the F–T sperm. In the CON group sperm, a positive correlation was observed between N-LTS and L-CTS (*p* < 0.05). A negative correlation was observed between At-LTS, L-CTS (*p* < 0.05) and N-LTS (*p* < 0.01), as well as between Ah-LTS and N-LTS (*p* < 0.01). In the E-Se group sperm, a positive correlation was observed between L-CTS and Abs.-F (*p* < 0.01) and between Ah-LTS and At-LTS (*p* < 0.01). A negative correlation was observed between N-LTS and At-LTS (*p* < 0.01) and Ah-LTS (*p* < 0.01).

Table 4 reports the performance of the two pheasant groups inseminated with F–T semen pellets from males fed on the CON or E-Se diet. The fertility percentage is identical in the two groups, reaching 30% in both, and the resulting incubation process output resulted in 8-9 hatched chicks for every 100 eggs incubated.

**Figure 1:**
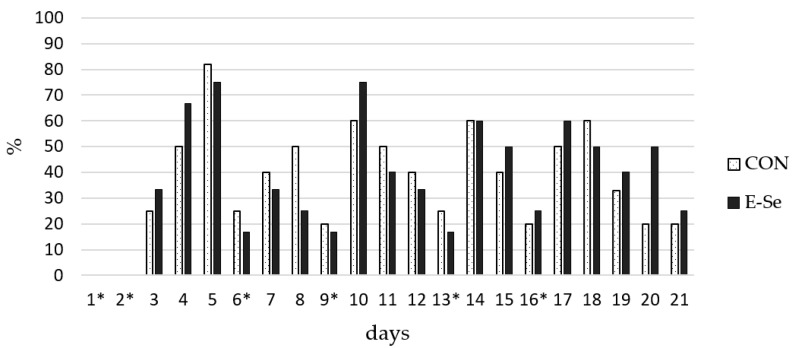
Fertility trend following AIs in common pheasants inseminated with 35 × 10^6^ of normal live thawed sperm (NLTS) from males fed on a standard commercial diet for breeders (CON) or the same diet enriched with E-200/Se-0.3 (E-Se). * = day on which the AI was performed.

Figure 1 illustrates the evolution of the percentage of fertile eggs following AI with F–T semen. Bell-shaped trends were observed. In both groups, an increase in the percentage of fertile eggs is observed on the day following each AI (indicated by asterisks). After the first and second consecutive AIs, both groups show a peak in fertility rate three days later. Thereafter, peaks are observed the day after AI in the E-Se group, whereas this trend only commenced in the CON group after the fourth AI. Longer fertility persistency is observed in the final bell for the E-Se group. No significant difference was observed in the daily fertility rate between the CON and E-Se groups (*p* > 0.05).

## 4. Discussion

The poultry industry has been using the massage technique for semen collection ever since it was first described by Quinn and Burrows [29]. Since its introduction, the technique has been adapted for use in non-domestic birds, and it continues to be the most common method used for semen collection [42]. It has even been reported possible to collect semen from uncooperative birds using this method, but the samples collected tend to be of poorer quality [42]. Whilst we can confirm the last statement from our experience with pheasants and chickens, our policy is to only collect semen from cooperative birds.

Here, for the first time, we describe our massage method for the collection of pheasant semen, which differs significantly to the one reported by Quinn and Burrows [29] as well as that by Gee et al. [42], since we never force the male bird to produce semen or the female to evert the cloaca. In fact, by permitting the birds to move as freely as possible, our method permits the collection of an abundance of good quality ejaculate the majority of the time. Considering that the first important step in the achievement of AI success is semen collection, this aspect is of extreme importance. On the contrary, the disadvantage of this technique is that it requires advanced bird hander experience and dexterity.

The ejaculate volumes observed in this study are consistent with our previously reported data for male pheasants of the same reproductive age, for which volumes ranged between 106 and 140 µL [36,41,43]. Males fed the E-Se diet in this study produced more abundant ejaculates compared with birds in the CON group (138 vs. 93 µL). This result contradicts our previous findings in which males fed the standard diet produced higher ejaculate volumes [44]. The positive influence of the diet enriched with E-Se on the sperm concentration agrees with the results a previous study [45]. Regarding the sperm concentration, it seems clear that diet is a key factor affecting cell number, as also reported for other species, such as roosters [46,47,48] and ganders, for which changes in diet were also shown to be capable of improving the volume and viability of the ejaculate [49]. Nevertheless, it is important to highlight that other factors (e.g., breeding conditions, health status, diet, environmental conditions, genotype, etc.) may also influence the production of high concentrated ejaculates, as evidenced by studies in which sperm concentrations per mL varied across years: 9.5 × 10^9^ in 2007, 12.5 × 10^9^ in 2008 [36], 10–12 × 10^9^ in 2009 [27,41], 5 × 10^9^ in 2010 [44] and 7.3–8.45 × 10^9^ in 2012 [45]. Comparing fresh semen parameters from birds belonging to the E-Se group in this study (9.11 × 10^9^/mL) with other pheasant species, we can note that the mean sperm concentration is more than 7.6-fold and 1.4-fold smaller in *Pucrasia macrolopha* and *Syrmaticus mikado*, respectively [50], and the average ejaculate concentration in the *Tragopan caboti* [51] is more than 3.2-fold and 3.9-fold smaller compared with our results for the CON and E-Se groups, respectively.

The fresh sperm viability percentages observed here lie within the 81–90% range reported in previous studies [27,36,43]. It seems that sperm survival remains quite stable regardless of external factors, whereas sperm concentration is more susceptible to influence by external factors. The sperm viability did not differ between the dietary groups for either fresh or F–T sperm. The viability data for F–T sperm agreed with previous data on common pheasants [43] as well as *Polyplectron emphanum* [50].

No difference was observed in fresh sperm mobility between the dietary groups. Similarly, Marzoni et al. [44] did not observe any differences in sperm motility between pheasants fed diets characterised by the same E-Se ratios (E-40/ Se-0.1 and E-200/ Se-0.3) as used in this trial. Sperm mobility is determined as the capacity of a sperm to accomplish a forward movement through a viscous medium [39], and this movement is measured by changes in the absorbance value. As expected, the freezing process decreases the mobility value, an 85% reduction compared with that achieved by fresh material. In fact, the consequences of oxidative damage occurring during the F–T process are numerous; it has been associated with the disruption of mitochondrial activity, the enhanced efflux of intracellular enzymes and damage to axonemal proteins. All these events result in the loss of the sperm motility [52]. The mobility of the F–T sperm was not affected by the diet. This result was unexpected considering that in the F–T E-Se semen a higher number of normal cells was observed, as was a positive correlation between cell viability and mobility. However, although a more complex scenario obviously occurs in vivo, the reliability of this “mobility test” was confirmed by the in vivo results of this trial since the fertility rates of the two dietary groups were identical.

The number of normal sperm cells after the F–T process varied between the dietary groups, with a higher number being detected in E-Se sperm. As expected, the F–T process brought about a large decrease in sperm number relative to the fresh sperm. In chickens, a decrease in the number of normal sperm after in vitro storage was associated with a decrease in the total lipid content of semen [9,53]. This occurs due to the high proportion of polyunsaturated fatty acids, which renders the avian sperm membrane susceptible to lipid peroxidation; thus, a decrease in the concentration of polyunsaturated fatty acids takes place [53]. Studies on the fatty acid composition of pheasant sperm have reported relatively low percentages of PUFAs (19–21%) and total UFAs (37–49%) [54] compared with other avian species such as the chicken (51 and 68%, respectively) [55], and similar levels compared with turkey sperm stored for one hour: 33 and 51%, respectively [56]. Throughout the F–T process, the lipids of sperm membranes and subcellular organelles are affected not only by the lipid peroxidation but also by their specific temperature phase transition, which differs from that of the water [9]. In this study, in the F–T sperm, the higher percentage of normal cells in the E-Se group compared with the control group (54 vs. 37%) might be the result of a more resistant and performing cell membrane. For example, in ruminants, disorders in the sperm cell antioxidant system during cryopreservation and the activation of L-amino-acid oxidase in defective or dead cells contribute towards a higher production of ROS, resulting in sperm plasma membrane damage [52,57,58]. In fact, high amounts of ROS induce the oxidative attack of PUFAs, resulting in the formation of toxic by-products such as malondialdehyde. This lipid assault, in conjunction with the conformational changes of the plasma membrane during the F–T process, contributes to the loss of membrane integrity and the disruption of its permeability properties [52,59].

Fractures were the most common injuries detected in the F–T sperm. The antioxidant-enriched diet appeared to exert its protection effect on the head-midpiece and tails of E-Se sperm, as revealed by comparing the damage incurred with that seen in the CON sperm. During the cryopreservation process, the sperm membrane integrity may be compromised due to alteration of the membrane fluidity, changes to the asymmetry of the phospholipid bilayer, and therefore its functional condition [52,59], and this is related to the membrane’s susceptibility to lipid peroxidation [13]. A higher susceptibility to oxidation has been associated with a decrease in cytoplasm volume, which is, at the same time, related to a reduced amount of antioxidant enzymes [60]. In fact, the antioxidant systems of cells include the natural fat-soluble antioxidants, such as vitamin E. Antioxidant enzymes are also involved, such as, for example, glutathione peroxidase (GSH-Px4), and selenium is an integral cofactor of this enzyme [13]. Additionally, the interaction between these two elements (vitamin E and selenium) may increase the production of GSH-Px4 [13,22]. The importance of Se in the diets of male birds was also reported for 14-week-old roosters, in which a dietary deficiency of this element retarded semen production by seven weeks and resulted in low relative testis weights, a high percentage of abnormal sperm in the ejaculates and a higher number of abnormalities in the sperm head midpiece [47]. In a study of male pheasants fed the same basal diet as used in the present study, the n-6/n-3 fatty acid ratio of sperm membranes was found to be lower in the group receiving the E-Se enriched diet (E-200/Se-0.3) [54]. According to another study in the chicken, diets rich in n-3 PUFA are associated with a higher requirement for vitamin E [61]. In the present study, the higher number of abnormal sperm cells in the CON group animals might have correlated with a possible difference in the plasma membrane composition, due to greater protection generated by the higher level of vitamin E.

The semen pH observed in this study did not differ between the two dietary groups and was consistent with the values reported in previous reports on pheasants (7.5–8.5) [45,50,51]. Considering pH values previously reported for pheasant semen (7.76–8.7) [27,43] and those in the chicken (6.8–7.85) [62,63], it appears that pheasant semen tends to be more alkaline, while that of the chicken remain slightly acid neutral. Indeed, large variations in pH are not expected considering the crucial nature of this physiological parameter [62]. Consequently, in the preparation of diluents, the pH should specifically be adapted for pheasants. Nevertheless, according to Saint Jalme et al. [50], increasing the diluent alkalinity did not improve sperm viability in a pheasant species characterised by a particularly alkaline semen. In the present study, a high positive correlation was observed between pH and At-LS and between pH and Ah-LS in the fresh sperm of both groups, suggesting that higher pH values render the environment less ideal for these cells, even if a positive correlation between pH and N-LS was seen in the sperm from pheasants from the CON.

Studies have reported accelerated lipid peroxidation to occur in abnormal sperm [64]. Furthermore, lipid peroxidation plays a role in regulated cell death [65], and an increase in the pH is related to a rise in the number of dead sperm [66]. In this study, no correlation was observed in the fresh semen between the viability of cells and the pH. Another study using a different pheasant species observed that higher pH values in fresh semen correlated with lower survival rates following the F–T process [50]. The positive correlations observed between the normal and abnormal cells in the fresh sperm of both groups in the present study was an interesting finding, and this correlation became negative in the F–T sperm, emphasizing the effect of the F–T process.

The egg weights observed in the present study are consistent with the mean data reported by other authors [67,68]. On the contrary, the mean egg weight in this study (33–34 g) was greater than those found in a previous study in which females were fed a vitamin E-enriched diet (E-200; 31.19–32.25 g) [69]. In natural mating, egg weight has no impact upon the fertility rate, but an effect of egg weight has been reported by other authors on egg hatchability, where eggs heavier than 35.99 g were less likely to hatch [67,68]. In this study, the egg weight had no influence on the fertility or the hatchability.

As expected, egg production did not differ between groups, since all females received the same feed. The egg production rate in the present study ranged from 85% to 88% and was higher compared with the 70% reported for females fed on diets enriched with vitamin E over a 4–12-week laying period [69]. This suggests that either the present study was performed at the time of maximum egg production or that the egg-laying performance of these females was actually better. Other authors have reported egg production rates of 64 and 74% in 1- and 2-year-old pheasants, respectively [70].

After one year of cryopreservation, the fertilising capacity of the F–T sperm had dropped to 30% in both dietary groups; considering the low insemination doses of only 35 × 10^6^ NLTS at 3–4-day intervals, this outcome is very promising. As expected, a substantial lower fertility rate was observed compared with the 83% obtained in pheasants inseminated once a week with a dose of 150 × 10^6^ fresh sperm, and the 40% fertility rated observed for 24-h stored sperm [71]. Comparable fertility values (26–37%) for F–T sperm have been reported under similar AIs conditions [27,45]. In the Combatiente chicken, doses of 500 × 10^6^ F–T sperm had a reported fertility of just 9.4% [72], whereas 87% fertility has been achieved in the turkey with doses of 400 × 10^6^ F–T sperm [73]. Increasing the sperm dose in the above-described AI protocol for pheasants would probably result in higher fertility percentages. The fertility trend observed after AI was similar in both female groups: the initial increase in fertility rate was gradual but was then followed by a sharper rise. Finally, the decrease in fertility rate tended to be slower for F–T sperm obtained from birds in the E-Se group.

Overall, no difference was observed in the reproductive performance of the two female groups inseminated with F–T sperm obtained from CON or E-Se birds. This result is supported by the finding that the mobility of the sperm cells did not differ between the two dietary groups either.

## 5. Conclusions

Dietary supplementation with α-tocopheril-acetate and selenomethionine improved the number and integrity of F–T sperm, resulting in a higher number of insemination doses. However, in addition to including antioxidants in the birds’ diet to protect cells from peroxidative damage, antioxidant inclusion in the extender might also help improve cell performance in vitro.

Doses of only 35 × 10^6^ live normal thawed sperm per AI generated fertility rates of 30%. The method of freezing sperm in pellets combined with hotplate thawing is an effective method for cryopreserving performing cells in pheasants. Here, it was able to guarantee an incubation process output of 89 hatched chicks for every 100 eggs incubated. Further studies are needed to identify additional and/or alternative strategies for improving the cryopreservation of sperm in pheasants.

## Figures and Tables

**Table 1:** Sperm qualitative parameters (means) measured in fresh and frozen–thawed (F–T) semen from common pheasants fed on basal commercial diet for breeders (CON) or the same diet enriched with E-200/ Se-0.3 (E-Se).

	CON	E-Se	SEM	*p*
Donors (n)	15	15		
Ejaculate volume (µL)	93.51	138.91	7.116	0.0010
pH	8.44	8.39	0.040	0.5200
Sperm concentration (×10^9^/mL)	7.48	9.11	0.180	0.0001
Sperm viability CS (%)	82.66	85.97	1.180	0.1712
Sperm mobility (A_550 nm_)	0.256	0.270	0.008	0.3308
Normal sperm LS (%)	77.49	82.44	2.370	0.2690
Abnormal sperm tail LS (%)	4.82	2.98	0.894	0.5220
Abnormal sperm head LS (%)	4.02	2.64	0.683	0.5826
Sperm viability CTS	25.48	25.05	1.803	0.8256
Sperm mobility FS	15.08	16.49	0.739	0.1849
Normal sperm LTS	37.13	53.78	2.255	0.0001
Abnormal sperm tail LTS	37.30	27.97	1.461	0.0007
Abnormal sperm head LTS	24.96	18.47	0.922	0.0002
**Sperm injuries**				
**Head**				
Bent sperm LTS	5.77	4.40	0.572	0.1300
Fracture sperm LTS	14.46	10.27	0.556	0.0001
Coiled LTS	0.09	0.01	0.028	0.1586
Swollen-detached LTS	3.78	3.38	0.402	0.9673
Knotted LTS	0.71	0.30	0.097	0.0623
Headless LTS	0.15	0.11	0.041	0.8398
**Tail**				
Looping LTS	8.89	8.23	0.667	0.6709
Fracture LTS	26.85	19.07	1.033	0.0001
Coiled LTS	1.57	0.67	0.278	0.0690

CS = relative to counted sperm; A_550mn_ = absorbance units read at 550 nm; LS = relative to live sperm; CTS = relative to counted thawed sperm; FS = relative to fresh sperm; LTS = relative to live thawed sperm.

**Table 2:** Correlations between the qualitative parameters of fresh sperm from common pheasants fed a basal commercial diet for breeders (CON) or the same diet enriched with E-200/ Se-0.3 (E-Se).

		Abs.	pH	SC	% L-CS	% N-LS	% At-LS	% Ah-LS
CON	Vol.	−0.0392	0.1320	0.1124	0.1845	−0.0616	−0.2269	−0.2597
	Abs.		−0.2702	0.1262	−0.1613	−0.0709	−0.5372	−0.1904
	pH			0.6967 **	0.0319	0.7936**	0.8304 **	0.8345 **
	SC				0.2262	0.7291*	0.4805	0.6508 *
	% L-CS					0.2674	−0.0561	−0.1771
	% N-LS						0.7417 **	0.8577 **
	% At-LS							0.8907 **
E-Se	vol.	−0.5107	0.3183	−0.2562	0.4277	0.1381	0.5683	0.4641
	Abs.		0.2816	0.1191	−0.6783	0.0466	−0.0267	−0.0677
	pH			−0.0735	−0.0068	0.7449	0.6339 *	0.7353 **
	SC				0.4146	−0.2801	−0.3106	−0.4873
	% L-CS					0.0342	−0.1407	−0.1578
	% N-LS						0.5475 *	0.7767 **
	% At-LS							0.9125 **

Abs = absorbance read at 550 nm to verify the sperm mobility by the Accudenz test [39]; SC = sperm concentration (×10^9^/ mL); % L-CS = live sperm rel. to counted sperm; % N-LS = normal sperm rel. to live sperm; % At-LS = abnormal tails rel. to live sperm; % Ah-LS = abnormal heads rel. to live sperm; * *p* < 0.05; ** *p* < 0.01.

**Table 3:** Correlations between the qualitative parameters in frozen–thawed (F–T) sperm from common pheasants fed a basal commercial diet for breeders (CON) or the same diet enriched with E-200/ Se-0.3 (E-Se).

		% L-CTS	% N-LTS	% At-LTS	% Ah-LTS
CON	% Abs.-FS	0.1836	0.3605	−0.3378	−0.2160
	% L-CTS		0.3880 *	−0.4237 *	−0.1221
	% N-LTS			−0.9552 **	−0.6121 **
	% At-LTS				0.3737
E-Se	% Abs.-FS	0.8470 **	−0.0372	0.0142	0.0269
	% L-CTS		−0.3258	0.2442	0.3602
	% N-LTS			−0.9664 **	−0.9279 **
	% At-LTS				0.8222 **

Abs.-FS = absorbance rel. to fresh semen read at 550 nm to verify the sperm mobility by the Accudenz test [39]; % L-CTS = live sperm rel. to counted thawed sperm; % N-LTS = normal sperm on live thawed sperm; % At-LTS = abnormal tails rel. to live thawed sperm; % Ah-LTS = abnormal heads rel. to live thawed sperm; * *p* < 0.05; ** *p* < 0.01.

**Table 4:** Egg laying, fertility and hatchability in common pheasants inseminated with frozen–thawed (F–T) semen pellets from males fed a basal commercial diet for breeders (CON) or the same diet enriched with E-200/ Se-0.3 (E-Se).

		CON	E-Se	*p*
Females	(n)	20	20	
Eggs laid		356	368	
Egg weight	(g)	33.83 ± 1.93	32.94 ± 3.21	0.073
Egg laying	(%)	84.76	87.62	0.615
Fertility		30.34	30.44	0.982
HFE		29.63	28.57	0.343

HFE = Hatchability, calculated as percentage relative to fertile eggs. Values are expressed as means ± sd.

## Data Availability

The data presented in this study are available on request from the corresponding author.
